# Delivering psychosocial support to family caregivers of cancer patients: Insights from Iranian psychosocial oncology professionals and family caregivers highlighting the need for change

**DOI:** 10.1017/S1478951525100618

**Published:** 2025-08-15

**Authors:** Baharan Ghavami, Fariba Zarani, Ladan Fata, Mohammad Reza Sharbafchi, Jacqueline Bender

**Affiliations:** 1 Department of Psychology and Educational Studies, Shahid Beheshti University, Tehran, Iran; 2 Department of Supportive Care, Princess Margaret Cancer Centre, University Health Network, Toronto, ON, Canada; 3 Dalla Lana School of Public Health, University of Toronto, Toronto, ON, Canada; 4 Education Development Centre, Iran University of Medical Sciences, Tehran, Iran; 5 Department of Psychiatry & Behavioral Sciences, Khorshid Hospital, Isfahan University of Medical Sciences and Iranian Cancer Control Center (Macsa), Isfahan, Iran; 6 Institute of Health Policy, Management, and Evaluation, University of Toronto, Toronto, ON, Canada

**Keywords:** Cancer, digital health, family caregivers, oncology, psychosocial oncology, psycho-oncology, psychosocial support

## Abstract

**Objectives:**

Supporting a family member with cancer poses significant challenges for family caregivers, who have unmet supportive care needs. Psychosocial oncology professionals (PSOP) are often the primary source of support for cancer caregivers in Iran. Given the lack of supportive care resources, innovative strategies are needed to support caregivers. This study explores the views of PSOP and caregivers regarding the challenges, potential solutions, and the role of digital technologies in supporting caregivers.

**Methods:**

Employing a qualitative descriptive design, we conducted individual interviews and focus groups with 30 participants (15 PSOPs and 15 caregivers), recruited from five settings in Tehran, Iran(2023-2024). All sessions were audio-recorded, transcribed verbatim, and analyzed using thematic analysis.

**Results:**

PSOP identified challenges in delivering psychosocial care to caregivers , including inconsistency, uncertainty, and fragmented use of technology. Their recommendations included flexible psychosocial care via blended multi-modal digital technologies, professional development opportunities, and formal recognition and integration within the oncology setting. Caregivers experiencing frustration with the healthcare system expressed a need for family-centered care, flexible psychosocial care, and organized peer support networks.

**Significance of results:**

Current psychosocial care in Iran is insufficient and misaligned with the preferences of PSOP and caregivers. PSOP and caregivers advocate for flexible psychosocial care through blended digital strategies. Public health strategists in Iran, as a low-resource setting with a family-centered context, should optimize resource utilization by prioritizing the training of PSOP, developing blended digital interventions, and leveraging trained peers to provide navigation and support to families, thereby easing the PSOP workload.

## Background

An informal caregiver is an unpaid family member or friend providing care to someone with whom they have a personal relationship (National Cancer Institute, 2025). There is a substantial body of research in Iran and globally on the high levels of distress experienced by cancer caregivers, the high burden of unmet supportive care needs (Hart et al. 2022; Pan and Lin, 2022; Rezaei et al. 2024) and their need for psychosocial support (Ahn et al. 2020). Despite the development of various supportive care interventions and the increasing number of cancer caregivers, little progress has been made in supporting them. A 2023 systematic review and meta-analysis showed that psychosocial interventions have a small effect size in reducing cancer caregivers’ burden, highlighting the need for more accessible and innovative services (Secinti et al. 2023). Since caregiving is rooted in culture (Kristanti et al. 2019), Iranian family caregivers see illness as a family problem (Hamedani et al. 2022), requiring culturally sensitive interventions (Secinti et al. 2023).

Psychosocial oncology professionals (PSOP) are mental healthcare providers who address the psychological, emotional, and social issues that arise for cancer patients and their loved ones (Canadian Association of Psychosocial Oncology, 2010). PSOP have a vital role in caring for people experiencing cancer-related distress, and many caregivers in Iran seek support from them. However, there are limited healthcare resources to meet the needs of the growing number of cancer survivors, leading to overwork (2019), necessitating innovative strategies to support cancer caregivers (Dhumal et al. 2023).

Virtual care and digital health psychosocial interventions may overcome some of these challenges and offer a promising way to reach and provide psychosocial care to cancer caregivers. In resource-constrained settings, maximizing existing resources through innovative, cost-effective technologies is crucial (Balogh et al. 2017). Additionally, the growing familiarity of people in low- and-middle-income-countries, like Iran, with digital technologies (Webb et al. 2022) offers opportunities to create virtual supportive care resources. Technology-based psychosocial interventions that include psychotherapy, psychoeducation, and skill training enhanced caregivers’ health-related quality of life and alleviated their depression but had limited impact on their self-efficacy and coping (Low et al. 2024). A systematic review and meta-analysis (Li et al. 2022) suggested integrating tailored technology-based interventions into routine psychosocial oncology care. PSOP have a desire to use video consultations (Schuster et al. 2024), and cancer caregivers have shown interest in web-based (Applebaum et al. 2018) and telephone support tools (Heckel et al. 2018). Yet, the preferences of Iranian PSOP and caregivers on technology-based interventions are underexplored. Given the high rate of Internet access and use in Iran (Keramatikerman et al. 2024), innovations in virtual care and digital health may offer a potential strategy to provide psychosocial care to cancer caregivers that aligns with their shared caregiving culture.

Little is known about the views of PSOP and cancer caregivers regarding the provision of psychosocial care to cancer caregivers and the role of digital technologies in Iran. Thus, the purpose of this study was to examine the views of PSOP and caregivers regarding the challenges and solutions to enhance psychosocial support for cancer caregivers, with a focus on the role of digital technologies in facilitating care delivery.

Unlike most studies of cancer caregivers, which have been limited to partners of cancer patients (Köhle et al. 2021), our study examines the views of a diverse range of caregivers with varying relationships to the patient (e.g., spouse, adult child, and extended family). The study findings will enhance our understanding of the challenges and potential solutions for the psychosocial care of cancer caregivers in low-resource, shared caregiving culture settings.

## Methods

### Study design

We conducted a qualitative descriptive study (Sandelowski 2000) using semi-structured individual interviews and focus groups to obtain the perspectives, experiences, and needs of PSOP and caregivers in supporting Iranian caregivers. We selected a qualitative descriptive design as it aligned with our aim – to collect PSOP and caregiver perspectives on challenges and solutions to enhance psychosocial care. This approach allowed us to stay close to the data with minimal interpretation, unlike other qualitative methods that aim to develop theory or uncover deeper meanings.

Interviews and focus groups were undertaken by the principal researcher (BG), a PhD candidate in Health Psychology. Qualitative thematic analysis (Braun and Clarke, 2006) was used to analyze the data, and the Consolidated Criteria for Reporting Qualitative Studies (COREQ) (Tong et al. 2007) were used to report the findings. The transcripts were coded by BG using a codebook collaboratively developed with the supervisory team (FZ, LF, JB). The analysis and reporting were led by BG in constant consultation with the supervisory team.

### Ethical considerations

This study was approved by the University of Shahid Beheshti Research Ethics Board (IR.SBU.REC.1402.010). Informed verbal and written consent was obtained from all participants.

### Inclusion and exclusion criteria

To be eligible to participate in this study, PSOP had to: 1) be a counselor, clinical psychologist, or health psychologist with a background in psychology or counseling; 2) have experience working with cancer patients and/or their caregivers; 3) be currently or formerly practicing in a hospital or private practice setting.

Cancer caregivers had to: 1) be an informal/unpaid caregiver of a patient with any type and stage of cancer, and 2) have a relationship with the patient, such as being a family member (e.g., spouse, parent, sibling) or a friend. Caregivers were excluded if they had lost their loved ones within the past month and were actively grieving.

### Setting and recruitment procedures

Data collection was conducted over a one-year period, from July 2023 to July 2024, and participants were recruited from five settings in Tehran, Iran.

PSOP were recruited from Macsa Psychosocial and Palliative Care Clinic at the Iranian Cancer Control Center in Fatima Public Hospital, the Adolescent and Young Adult Oncology Clinic in Ali Asghar Public Hospital, the Department of Psychology and Educational Studies at Shahid Beheshti University, and two private clinics providing clinical health psychology services. PSOP who worked in hospital settings provided both inpatient and outpatient psychosocial care to patients and caregivers. Caregivers were recruited from Macsa and one private clinical health psychology clinic.

A purposive sampling strategy was used to recruit a maximum variation sample of PSOP and caregivers (Sandelowski 2000), considering factors such as years of experience and service settings for PSOP, as well as cancer type and stage, socioeconomic status, and caregiver-patient relationship for caregivers.

Of the 19 PSOP contacted, 15 agreed to participate. PSOP who were recruited from Macsa Clinic were first approached by a clinician at Macsa, who explained the study, and the researcher (BG) obtained their consent to participate in the study. PSOP recruited from other settings (Adolescent and Young Adult Oncology Clinic in Ali Asghar Public Hospital, Department of Psychology and Educational Studies at Shahid Beheshti University, and two private clinics) were approached by the researcher (BG). The first focus group, consisting of seven PSOP, was held in-person, and the second focus group, consisting of five PSOP, was held virtually through Skype video conference. Two individual interviews were conducted in-person and one virtually via phone for PSOP who were unable to attend focus groups.

Of the 17 caregivers contacted, 15 agreed to participate. Caregivers were approached by the researcher (BG) when they were accompanying patients during regularly scheduled appointments at the Macsa palliative care outpatient clinic or during an inpatient stay. If they were interested and agreeable, the researcher (BG) obtained consent and scheduled the in-person interview at the clinic. Ten interviews took place in a private room at the Macsa clinic. The researcher (BG) also contacted caregivers from a private clinic who had agreed, through their clinician, to be contacted about the research study. Five caregivers, who were informed about the study through a private clinic, were interviewed virtually via phone.

### Data collection

Individual interviews were conducted with caregivers to allow them to discuss their views, experiences, and sensitive topics openly, while focus groups were conducted with PSOP to provide an opportunity to obtain a broad range of information about PSOP challenges and solutions in a group discussion (Sandelowski 2000). Individual interviews were also offered to PSOP who could not attend the focus groups.

The researcher (BG) moderated the focus groups and conducted the individual interviews. During the focus groups, all participants had equal opportunities to speak, and the researcher asked probing questions to facilitate the sharing process. While there were occasional differences in perspectives among participants, which is natural in group discussions, these did not escalate into conflict. In times of differing views, PSOP expressed their insights in a respectful manner and were willing to continue the discussion until reaching a consensus.

Focus groups with PSOP lasted 120 minutes, and each individual interview with PSOP or caregivers lasted 45 to 75 minutes. Demographic information for each participant was collected verbally just before the focus groups and interviews. All interviews and focus groups were audio-recorded and transcribed verbatim, and field notes were taken by the researcher (BG). Separate semi-structured guides for interviews and focus groups were developed for PSOP and caregivers based on the study objectives and literature review (Tables 1 and 2). These were reviewed and discussed with study team members.

**Table 1.** PSOP focus group and interview questions

Question	Probes
What has been your experience providing psychosocial care to the cancer caregiver population?	How is the quality of therapy sessions with caregivers?
What are your challenges in working with cancer caregivers?	What obstacles do you often encounter when working with caregivers that are less discussed?
How do you utilize digital/online services to support caregivers?	Can part of your responsibilities be delegated to digital technologies? If so, which aspects?
How do you think you can better meet the needs of caregivers?	What could have happened to make your interventions more effective and beneficial for caregivers?

**Table 2.** Caregivers interview questions

Question	Probes
As a caregiver, have you ever sought psychological support?	How was your experience seeking psychological support?
What are the primary challenges and obstacles you face in addressing your psychological needs?	What difficulties have you encountered in receiving such care?
In seeking support, do you often use the Internet for gathering information?	How would you prefer digital technologies to support you in your caregiving journey?
What improvements do you believe should be made to better meet your psychosocial needs?	–

We continued sampling to the point of saturation, at which new data no longer significantly contributed to the emergence of new themes (Ando et al. 2014). For PSOP, one focus group and three individual interviews were conducted, followed by a second focus group to ensure thematic saturation. The second focus group confirmed consistency with the previously collected data, and no new themes emerged. For caregivers, data saturation was reached by the 10^th^ interview; however, interviews were continued to ensure thematic saturation and confirm the consistency of emerging themes.

### Data analysis

Transcripts were managed using MAXQDA (v. 2020) qualitative data analysis software and analyzed following Braun and Clarke's thematic content analysis procedures (Braun and Clarke, 2006). After each interview and focus group, audio recordings were transcribed and reviewed repeatedly to familiarize the researchers with participants’ challenges and needs. Two separate code books were generated, beginning with coding the first PSOP focus group and interview transcripts, followed by caregivers’ interviews and the second PSOP focus group. Transcripts were coded into meaningful units containing relevant information, which were then analyzed and grouped into subcategories based on conceptual similarities. These subcategories were further classified into main categories called themes.

Constant comparison of data within and between the PSOP and caregivers helped identify connections and relationships between codes and themes. Codes were expanded to highlight overlapping concepts, similarities, and differences between the two participant groups. Discussions and feedback on codes and themes were provided by the supervisory team (FZ, LF, JB), and the data interpretation and analysis were cross-checked with them. The focus groups and interviews were conducted and analyzed in Farsi, and the codebook, findings, and quotations were translated into English.

## Results

### Sample characteristics

In total, 30 participants participated in this study. Of these 30 participants, 15 were PSOP and 15 were cancer caregivers. Of the 15 PSOP, most were recruited from public health settings (80%), and most were females (93.7%), with an educational background in Health Psychology (56.2%), a PhD degree (62.5%), and an average of 7.5 years of work experience in the field (40%).

Of the 15 caregivers, most were recruited from public health settings (73.3%), and most were females (80%) with a university degree (86%), caring for metastatic cancer patients (66.6%) either with leukemia or breast cancer (representing 26.6% each), and were either spouses, siblings, or children (representing 20% each) of cancer patients.

Characteristics of PSOP and caregivers are reported in Tables 3 and 4, respectively.

**Table 3.** PSOP characteristics

PSOP characteristics	N (15)	N (%)
Educational Background		
• Health Psychology	7	46.6
• Clinical Psychology	5	33.3
• Psychology	2	13.3
• Counseling	1	6.6
Degree		
• PhD	9	60
• PhD Student	5	33.3
• Master’s	1	6.6
Gender		
• Women	14	93.3
• Men	1	6.6
Years in practice		
• 5–10	6	40
• <5	5	33.3
• >10	4	26.6

**Table 4.** Caregivers characteristics

Caregivers characteristics	Categories	N (15)	N (%)
Gender	Women	12	80
	Men	3	20
Age	17-40	8	53.3
	40-63	7	46.6
Education	Undergraduate	7	46.6
	Graduate	6	40
	High school	1	6.6
	Illiterate	1	6.6
Employment	Employed	11	73.3
	Unemployed	4	26.6
Race/Ethnicity	Iranian	14	93.3
	Afghan	1	6.6
Patient’s cancer diagnosis	Leukemia	4	26.6
	Breast	4	26.6
	Brain	2	13.3
	Lung	2	13.3
	Bladder	1	6.6
	Uterine	1	6.6
	Skin	1	6.6
Patient’s cancer status	Metastatic	10	66.6
	Non-metastatic	5	33.3
Type of caregiving	Shared	12	80
	Sole	3	20
Relation to the patient	Spouse	3	20
	Sibling	3	20
	Child	3	20
	Parents	2	13.3
	Grandchild	1	6.6
	Niece	1	6.6
	Sister-in-law	1	6.6
	Aunt	1	6.6

### Findings

During the focus groups and interviews with PSOP, they described their challenges in delivering support to caregivers and their need to improve the current situation. Their challenges were categorized into three themes and six subthemes. PSOP identified inconsistency, uncertainty, and fragmented use of technology when working with cancer caregivers as the main challenges. To enhance their current situation, three potential solutions were proposed: flexible support, professional development opportunities, and formal recognition and integration into oncology settings. Caregivers described their challenges and needs in receiving psychosocial support. Overall, one theme and two subthemes emerged as their challenges. They expressed frustration with the healthcare system as a main theme. Their solutions were categorized into three themes: family-centered psychosocial care, flexible psychosocial care, and organized peer support networks. Themes, subthemes and representative quotes for PSOP and caregivers are summerized in Table 5 and 6, respectively.

**Table 5.** PSOP challenges and solutions

Challenges		
Themes	Subthemes	Quotes
Inconsistency	Missed appointment	“Caregivers often cancel last-minute or miss appointments due to their unpredictable responsibilities. I understand, but it disrupts our schedule and makes it difficult to follow-up.” (FG^†^, P7)
	Navigating workload demands within a shared caregiving model	“Sometimes the whole family is here [in the hospital]. I speak to the primary caregiver, and they ask me to talk to other family members too … but there is not enough time for everyone.” (FG, P1)
	Insufficient staff	“The number of PSOP has always been unmatched with the needs of patients. We should establish protocols for training and keeping PSOP in the system.” (FG, P13).
	Inpatient-based psychosocial care	“Providing psychological care to caregivers in inpatient is more challenging than in private clinics. In inpatient, we have to actively reach out to them.” (FG, P4)
		“Caregivers tell me they hate the hospital, streets, and regions close to it.” (FG, P15)
Uncertainty	Insufficient education, supervision, and scientific opportunities	“We are experiencing ambiguity, same as patients. When caregivers are in the hospital, I am unsure how often to check on them, whenever they want, or when I can? It’s a professional dilemma.” (FG, P2)
	Lack of culturally-based caregiver clinical practice guidelines	“We have clear guidelines for anxious or depressive clients, but psychosocial oncology and caregiver support are uncharted territory. There’s no framework – just figuring out visit frequency and boundaries with flexibility as we go.” (Interview, P9)
Fragmented use of technology in care delivery	–	“My colleagues and I have developed digital products, but they remain unpublished and ineffective. I’d be interested in using other’s tools for caregivers instead of starting from scratch. We need an R&D team – service delivery is chaotic.” (Interview, P10)

Solutions	
Themes	Quotes
Flexible support via blended digital multi-modal technologies	“Many services can be delivered digitally. We handle follow-ups and sessions by phone (FG, P1). Existential or death-related issues require face-to-face discussions (FG, P6). I conduct all couples therapy online (FG, P5). Children and adolescents sometimes cooperate well online, but not always (FG, P2). For highly anxious caregivers, online or phone sessions are ineffective – they need to be present for better management (FG, P8).”	
Professional development opportunities	“There is no professional supervision to guide us through this ambiguity. I consult my supervisor; although he is a psychologist, he lacks specialization in oncology. We need specialized education.” (Interview, P9)	
Formal recognition and integration of psychosocial care in oncology.	“A systemic issue is the lack of a culture involving psychologists in decisions and procedures. For instance, a physician may break the news of a cancer recurrence in a hospital hallway, then refer the patient to us when things are at their worst. What if they had involved us earlier, using our expertise.” (Interview, P9)	

† FG: Focus Group.

**Table 6.** Caregivers' challenges and solutions

Challenges		
Themes	Subthemes	Quotes
Frustration with the healthcare system	Financial challenges	“I am spending most of my time commuting between the pharmacy, insurance office, and hospital. Talking to other caregivers, it is clear we are all under financial pressure. It wears you down. Our quality of life has obviously taken a hit since cancer entered the picture.” (Wife, C2)
	Feeling of Disorientation	“I wasn’t prepared for caregiving. Nobody told me my mother would become more sensitive due to treatment or how to handle it. I jumped in unprepared. It was so difficult. I was young and knew nothing.” (Daughter, C3)

Solutions		
Themes		Quotes
Family-centered Psychosocial Care		“It’s not just me; the entire family requires psychological support. I’m not alone in this journey – my brother and sister-in-law need it too.” (Aunt, C1)
Flexible Psychosocial Care		“When I’ve got downtime, like waiting during chemotherapy or at night, I wish I could get some mental and educational support.” (Sister, C11)
Organized Peer Support Networks		“I hope there will be an organization that holds different programs for caregivers. Either entertainment or educational. Just us. We are the people who understand each other.” (daughter, C5)

#### PSOP challenges in delivering psychosocial care

***Theme 1. Inconsistency***: One of the challenges described by PSOP in providing psychosocial care for caregivers was that the support is not delivered on a regular and continuous basis. Inconsistencies arose from several factors, including missed appointments, navigating workload demands within a shared caregiving culture, insufficient staff, and inpatient-based psychosocial care.

*Subtheme 1. Missed appointments*: PSOP described a high rate of dropouts and last-minute appointment cancellations, which interrupted their workflow and reduced the therapeutic benefits for caregivers. One PSOP noted the closure of an outpatient caregiver psychosocial oncology clinic due to the high rate of no-shows and cancellations from caregivers.

*Subtheme 2. Navigating workload demands within a shared caregiving model*: PSOP reported being overloaded with patients’ psychosocial care, leaving insufficient time for caregivers. PSOP mentioned that in the shared caregiving model, sometimes a primary caregiver seeks support and then supports the rest of the family, while at other times, multiple caregivers seek support. This requires more effort, time, and energy to manage the family, exacerbating the existing workload.

*Subtheme 3. Insufficient staff*: all PSOP agreed that there is a misalignment between the needs of patients and caregivers and the available supportive resources, such as the number of PSOP in hospital settings. They reported that a lack of staff makes it difficult to follow up with caregivers while managing new cases. PSOP mentioned that they struggle not only with a lack of staff but also with staff inconsistency, meaning that new PSOP often leave after a short period in an oncology setting.

*Subtheme 4. Inpatient-based psychosocial care*: PSOP noted that maintaining consistency in psychosocial care in a hospital’s inpatient settings is more challenging than in outpatient clinics. According to PSOP, maintaining continuity of care, providing consistent support, and ensuring follow-up in inpatient settings was challenging, as patients and caregivers frequently transitioned in and out. One PSOP stated that care delivery to caregivers is limited to the duration of a patient’s hospital stay, and there is a lack of clear procedures for post-discharge. Additionally, most PSOP felt that hospitals do not create a secure and peaceful environment, which is a prerequisite for providing psychological support. PSOP believed that caregivers want to avoid the hospital and its associated memories.

***Theme 2. Uncertainty***: PSOP reported experiencing uncertainty in several aspects of providing care to caregivers, such as maintaining a balance between handling cancer-related issues and caregiver’s mental health disorders, given their limited time in supporting them, the frequency of follow-ups, boundaries of supportive care, how to integrate technology into their work, and how to manage inconsistency. PSOP stated that ambiguity can decrease their self-efficacy and confidence in working with caregivers.

According to PSOP's point of view, uncertainty stemmed from insufficient supervision and scientific opportunities in psychosocial oncology, as well as a lack of culturally-based caregiver clinical practice guidelines.

Subtheme 1. *Insufficient supervision and scientific opportunities in psychosocial oncology*: PSOP complained about the lack of specialized psychosocial oncology supervisors to seek professional support, as well as the absence of scientific networks and conferences to share knowledge and stay updated.

Subtheme 2. *Lack of culturally-based Caregiver Clinical Practice Guidelines*: PSOP needed practical guidelines for working with caregivers. They believed that while there are many guidelines in psycho-oncology, caregiver-specific guidelines are needed within a shared caregiving culture. They mentioned that caregivers should be assessed and screened alongside patients, as they play a vital role in the care journey.

***Theme 3. Fragmented Use of Technology in Care Delivery*.** PSOP highlighted the disorganized and fragmented use of technology. They noted the lack of integrated platforms, with each PSOP independently trying to incorporate technological tools into their workflow. This often involved creating multimedia resources, such as mindfulness audio guides, from scratch. They expressed frustration over the inefficiency of this process and believed better coordination was needed to avoid duplication. They emphasized the importance of commercializing, making digital interventions widely accessible, promoting existing resources, and developing new ones. These efforts could enhance cost-effectiveness and streamline intervention initiatives.

#### PSOP solutions for improved psychosocial care

***Theme 1. Flexible Support via Blended Digital Multi-modal Technologies***: PSOP emphasized the need for flexibility in delivering supportive care and recognized that digital technologies offer valuable tools for this purpose. They suggested that designing digital-based interventions is key to supporting caregivers without relying on in-person visits to hospitals. They found digital platforms useful for providing psychoeducational support.

PSOP believed that psychosocial care could be delivered both synchronously and asynchronously. Psychoeducational content could be offered asynchronously, allowing caregivers to benefit from support at their own time and pace. However, initial assessments, discussions about emotions, and group therapy are better suited for synchronous delivery. Follow-ups could be delivered either synchronously or asynchronously. PSOP stated that utilizing online tools facilitated care delivery to cancer patients and their caregivers. However, services should adopt a blended approach, as internet instability and poor connections in some regions present significant barriers, making online access unreliable for everyone.

***Theme 2. Professional Development Opportunities in Psychosocial Oncology***: PSOP were eager to access professional training, ongoing psychosocial oncology supervision, and opportunities to participate in scientific networks. They believed formal accreditation procedures should be established by health policymakers to enhance their profession, and psychosocial oncology training practicums should be developed. They specifically emphasized two areas for the practicum: learning a supportive approach with ethical decision-making and exploring the possibilities of technology. While PSOP acknowledged the promise of digital technologies, they were unsure how to effectively integrate them into their practices. They also believed digital literacy is essential in the digital age, especially post-Covid-19. They needed to understand the potential of technology and how to align it with their therapeutic approach.

***Theme 3. Formal recognition and integration of psychosocial care in oncology***: PSOP acknowledged progress in recognizing the importance of psychological care in chronic disease settings but noted there is still room for improvement. Psychosocial oncology needs better integration within the healthcare system and collaboration with other providers, such as oncologists and nurses. PSOP believes their involvement at different stages of the cancer journey – from breaking the diagnosis to survivorship or death – is beneficial.

They stated that being an embedded PSOP fosters multi-professional teamwork. PSOP noted that they currently take on the sole responsibility for supportive care; this should be shared with social workers, oncology nurses, volunteers, and peers. PSOP also highlighted the need to develop collaborative skills with other healthcare providers, noting that shared decision-making improves patient and caregiver outcomes.

#### Caregivers’ challenges in accessing psychosocial care

***Theme 1. Frustration with the healthcare system***: When asked about perceived barriers to accessing psychosocial care, caregivers expressed frustration with the healthcare system’s failure to meet their needs. They provided examples associated with this frustration, such as feeling ignored by healthcare providers when cancer news is shared with the patient. Caregivers believed they should be consulted, as they act as gate keepers in deciding whether the patient should be informed about their diagnosis. They described that they deserved a more empathetic attitude from the healthcare system, which does not offer sufficient informational, emotional, and financial support. Financial challenges and feelings of disorientation contributed to their frustration.

*Subtheme 1. Financial challenges*: Caregivers faced significant financial challenges during cancer treatment, including the cost of treatment, obtaining medications, dealing with insurance companies, and commuting to cancer hospitals, all of which added to their burden and anxiety. They believed that the system not only took no steps to mitigate these challenges but also did not understand or empathize with them. Financial concerns often took priority, leaving caregivers with limited time and motivation to seek psychosocial support. The high cost of private psychological care further limited accessibility, making it unattainable for many. Caregivers agreed on the need for financial aid resources to help alleviate these burdens.

*Subtheme 2. Feeling of Disorientation*: Caregivers reported feeling disoriented and confused after their loved one’s diagnosis because of insufficient support from the healthcare system. They expected the healthcare system to involve them more actively and better prepare them for this journey with more empathy and the provision of informational (e.g., nutrition, treatment, side effects, etc.) and emotional support. They reported that balancing caregiving and other life aspects was challenging, and they expected the system to help them find balance in their lives.

#### Caregivers’ needs for improved psychosocial care

***Theme 1. Family-Centered Psychosocial Care***: Caregivers viewed caregiving as a shared family responsibility, involving both immediate and extended family members. They were open to distributing caregiving tasks, believing it helped them perform better. Of the 15 interviewees, 12 had experienced shared caregiving and found it effective, while three solo caregivers, lacking support, expressed a desire for shared caregiving. Since they saw cancer as a family issue affecting everyone, they valued services that support the entire family or caregiving circle.

***Theme 2. Flexible Psychosocial Care***: Caregivers emphasized the need for accessible and reliable psychological support that fits into their daily tasks. Given their busy schedules, the demands of managing both personal and caregiving tasks, and the fatigue caused by the caregiving burden, they expressed a preference for receiving support through various methods tailored to their specific conditions. Additionally, they stated that at times during the caregiving journey, they felt reluctant to talk about their painful emotions, preferring to ignore and distract themselves. Therefore, they might not be ready to speak with PSOP yet still required some form of support. Caregivers suggested that using different platforms, such as mobile applications and both online and offline resources, could provide flexible support.

***Theme 3. Organized Peer Support Networks***: Most caregivers valued peer support; only one of them did not. Those who were willing to connect with peers reported positive experiences that enhanced their resiliency. They expressed interest in attending caregiver-specific events and activities organized by trusted organizations.

## Discussion

The findings of this study align with global initiatives to unify supportive cancer care by 2030 (Chan et al. 2024b). This underscores the need for policy initiatives that promote a team-based approach (a multidisciplinary teamwork involving coordinated care from a range of psychosocial and medical professionals), evidence-informed education, financial impact minimization, guideline-driven care, and dedicated supportive oncology services for families affected by cancer.

Addressing the challenges identified by PSOP and caregivers requires action at the macrosystem level, involving public health authorities and policymakers. Strengthening national policies and effectively allocating resources are essential to improving the accessibility and quality of psychosocial care. An international survey of psychosocial care for cancer survivors (Signorelli et al. 2024) highlighted the critical role of national policies in facilitating psychosocial care delivery across different income settings. As a low-resource country, Iran faces significant challenges in providing consistent and sufficient psychosocial support to cancer families. Caregivers’ overall perception of the healthcare system is one of frustration, which can act as a barrier to seeking psychosocial services. This frustration, stemming from unmet supportive needs, highlights the need for systematic improvements.

Although this study did not explicitly inquire about caregivers’ perception of their role, many viewed themselves as gatekeepers – a culturally rooted role aimed at protecting their loved ones by filtering the amount of information shared with them and being actively involved in medical decision-making. This finding aligns with a previous study (Nasrollahi et al. 2022) showing that Iranian family caregivers often request oncologists to allow them to control the disclosure of a cancer diagnosis; most prefer to be present during the delivery of such news, and over half wish to be informed before the patient. This may lead to conflict with healthcare providers’ practices, as not all prioritize caregivers’ involvement, resulting in caregiver frustration. This should be considered when communicating diagnoses with patients and their caregivers, and further research is needed to explore how the healthcare system can better navigate these cultural expectations.

Consistent with Cheng et al.'s systematic review (Cheng et al. 2022), caregivers felt disoriented after their loved one’s diagnosis, lacking both informational and emotional support. An international survey on global disparities in cancer supportive care (Chan et al. 2024a) reported that timely supportive care is the most important disparity, which aligns with caregivers’ perception of insufficient support and disorientation. PSOP acknowledged this challenge, citing limited time, staff, and resources as barriers to addressing caregivers’ needs. The lack of allied health providers is a global challenge in providing psychosocial care (Signorelli et al. 2024), which highlights the provision of digital services to support PSOP and enhance service delivery.

Despite caregivers’ willingness to receive support, they often find it inflexible, leading to inconsistency – a challenge faced by PSOP. Currently, synchronous psychosocial sessions are the only method for providing psychosocial care to caregivers. However, they should not be the sole resource for caregiver support, as this partly explains the inconsistency and inflexibility. Nissim and Hales (2023) indicate that a single intake session for caregivers, coupled with an introduction to available supportive resources, is a potential framework for supporting caregivers. In Iran, the lack of community-based supportive care resources – such as educational workshops, wellness programs, financial aid, support groups, and peer navigation – exacerbates this issue. Consequently, the responsibility of providing psychosocial care largely falls on PSOP.

Among various support resources, peer support offered by credentialed organizations is essential yet underutilized. Similar to Papadakos’ study (Papadakos et al. 2023), caregivers in this study wanted to connect with other caregivers who shared similar experiences and living conditions (Breuning et al. 2024). Support and navigation from other caregivers (Chan et al. 2023) can mitigate caregivers’ uncertainties and complement PSOP care. Organizing and training peers to provide support and navigation is a potentially affordable supportive care intervention in resource-limited regions (Chan et al. 2024a; Soto-Perez-de-Celis et al. 2021) offering benefits for both navigators and peers (Vodermaier et al. 2023). Despite their growing recognition as a vital aspect of supportive care in low- and-middle-income countries (Cabanes et al. 2022), their implementation in Iran is rare. Further exploration is needed to adapt such peer support programs to Iran’s cultural and linguistic context.

Care delivery in Iran is further shaped by the cultural construct of shared caregiving. While shared caregiving has meaningful and spiritual value for caregivers, healthcare systems are not equipped to accommodate the needs of entire families. This increases PSOP’s workload, requiring additional time and resources to support families, especially in Iran, a resource-constrained setting. Healthcare systems must acknowledge shared caregiving and incorporate it into clinical practices by developing family-centered programs (Kristanti et al. 2019).

To address these challenges and make psychosocial care more accessible to families, PSOP and caregivers advocate for flexible support through blended digital interventions. Blended support enables caregivers to receive assistance without hospital visits, which can be a triggering environment. Dhumal et al. (2023) also recommend flexible support for its accessibility, reduced no-show rates, and fewer appointment cancellations, addressing a key challenge identified by PSOP. Despite the promise of blended technologies, their effectiveness in supporting caregivers remains underexplored. Although both stakeholders emphasize the importance of diverse support options to allow caregivers to access services when needed, they are not provided effectively, with PSOP mentioning fragmented utilization. For Iran, as a developing country, this marks a starting point for developing and adapting these technologies in healthcare settings.

Innovative technologies can be implemented to optimize available resources. Given the interest from private vendors, designing and implementing blended psychosocial interventions – including educational modules, online support groups, and peer navigation programs – can enhance caregiver support and ensure sustainable care delivery. Collaboration between PSOP and technology developers can help overcome these barriers, decrease the fragmented utilization of technology, and advance the adoption of digital psychosocial support services.

In alignment with Schuster et al. (2024), PSOP in this study recommended delivering emotion-focused interventions synchronously and psychoeducational content asynchronously. There is a need to digitalize existing psychosocial interventions and tailor them to caregivers’ unmet needs and burdens. However, PSOP lack the digital skills to integrate these technologies into their services; thus, they advocate for digital health literacy to be incorporated into their training practicum.

Although we did not specifically inquire about PSOP job-related challenges, they shared difficulties they faced in their profession. Resource limitations affect both caregivers and PSOP. According to the job demand-resources model, high job demands and inadequate resources are associated with higher burnout (Bakker and Demerouti, 2007). Iranian PSOP are at risk of burnout due to inadequate training and supervision. Inadequate psychosocial oncology supervision in Iran’s educational and health system fails to prepare PSOP properly, aligning with other studies showing PSOP desire for ongoing training (Wiener et al. 2012) to maintain their effectiveness in the emotionally intensive field of cancer care (2020). Role ambiguity is common among PSOP (Kocańda and Jabłoński, 2023; Kracen et al. 2020) due to inadequate training and supervision.

The educational system should consider pathways to empower PSOP, including continuing education, online and offline training modules, AI-assisted role plays, and proper clinical supervision. It is suggested that cancer centers consider integrating psychosocial oncology training into job responsibilities, compensation, and working hours (Raque-Bogdan et al. 2019). Not only PSOP, but also other members of the multi-disciplinary team – such as nurses, social workers, and oncologists – could benefit from learning how to provide basic psychosocial care earlier in their education. This would better prepare them to support families affected by cancer. In higher-level education for psychologists, competency-based programs such as postdoctoral fellowships in psychosocial oncology prepare trainees for inter-disciplinary work through clinical supervision. Interprofessional cooperation decreases uncertainty, leading to holistic views and ensuring the necessary services are provided for caregivers (Hamedani et al. 2023). This is in line with PSOP's suggestion of embedding psychosocial care within the circle of care and multidisciplinary teamwork to reduce role conflicts and increase communication between different specialties to better support families affected by cancer.

Participating in national and international professional events can increase professional cohesion and reduce isolation (Wiener et al. 2012), which is particularly significant for a country like Iran, which has a greater need to learn and limited resources to approach. Moreover, the implementation of supportive care guidelines is a major disparity in low- and-middle-income countries (Chan et al. 2024a), while they are essential to address PSOP’s uncertainty. Clinical oncology guidelines highlight the importance of culturally informed and sensitive care (Bergerot et al. 2024), aligning with PSOP’s request. The psychosocial oncology field has room for growth in Iran, particularly through formal ongoing training, professional networking, and culturally tailored clinical practicums.

## Limitations

This study has important limitations. Among PSOP, we recruited those with a background in psychology and counseling, as they are the primary providers of psychosocial oncology care. Consequently, our sample did not include social workers, psychiatrists, or spiritual counselors. The findings may not be representative of all mental healthcare providers who work in oncology settings, and further research would need to consider the views and needs of diverse psychosocial care providers in Iran. Additionally, only one PSOP participant was male, so to increase gender inclusivity, more data is needed. While efforts were made to include a diverse range of family caregivers, Iran’s various sub-cultures were not fully represented in the study sample. Therefore, caution should be exercised when generalizing the findings to all Iranian family caregivers. The coding and data analysis were conducted by a single researcher (BG), which may have introduced potential bias. Lastly, although strategies were used to preserve trustworthiness in translating findings and quotes from Farsi to English through forward and back translation, participants’ answers, meanings, or some context may have been unintentionally lost in translation.

## Clinical and research implications

Future clinical and research efforts should focus on developing blended services and support resources for caregivers. Research should assess the value and cost-effectiveness of such interventions for caregivers, PSOP, and the broader healthcare system. Clinical practice should prioritize creating online and offline education and wellness programs through partnerships with industry partners in private and public health settings, enriching the supportive care toolkit for caregivers.

These findings are useful in designing training and supervision for mental health professionals interested in psychosocial oncology. The American psychosocial oncology core-curriculum (Wells-Di Gregorio et al. 2022) should be adapted and culturally customized for the Iranian context. Moreover, due to the lack of training and supervision in PSOP, further investigation into the prevalence of burnout among Iranian PSOP is needed.

## Conclusion

This study underscores the need to improve psychosocial support for cancer caregivers in Iran, as current services are insufficient and misaligned with the preferences of PSOP and caregivers. Psychosocial services should extend beyond hospital settings by integrating community-based and online psychoeducational resources to complement the support provided by PSOP. A blended approach – combining in-person and digital interventions – can provide flexibility and accessibility while maintaining essential human interaction. It is recommended that private sectors with available funding resources support public health initiatives by participating in the development of blended interventions. Trained peers may play a helpful role in navigating families through their cancer journey, alleviating uncertainties, and providing an additional layer of support. Additionally, formal structured training and systemic changes are necessary to better support PSOPs’ career development. By implementing these strategies, healthcare systems in Iran can foster a more inclusive and accessible psychosocial care delivery framework.

## Supporting information

10.1017/S1478951525100618.sm001 Ghavami et al. supplementary material Ghavami et al. supplementary material
