# Supplementary material for: Delivering psychosocial support to family caregivers of cancer patients: Insights from Iranian psychosocial oncology professionals and family caregivers highlighting the need for change
Source: Palliat Support Care. 2025 Aug 15;23:e141. doi: 10.1017/S1478951525100618 (PMC13166713; doi:10.1017/S1478951525100618)
Supplement: Ghavami et al. supplementary material [file S1478951525100618sup001.docx]

Consolidated Criteria for Reporting Qualitative Research (COREQ) checklist:

| No | Item | Description | Description in this study |
| --- | --- | --- | --- |
| **Domain 1: Research team and reflexivity** | | | |
| Personal Characteristics | | | |
| 1 | Interviewer/facilitator | Which author/s conducted the interview or focus group? | The first author (BG) conducted the interview. |
| 2 | Credentials | What were the researcher's credentials? E.g. PhD, MD | First author: MA, PhD Candidate  Second author: PhD  Third author: PhD  Fourth author: PhD  Fifth author: PhD |
| 3 | Occupation | What was their occupation at the time of the study? | First author: Graduate student, clinical health psychologist  Second author: Associate professor, clinical health psychologist  Third author: Assistant professor, clinical health psychologist  Fourth author: Assistant professor, psychiatrist  Fifth author: Associate professor, scientist |
| 4 | Sex | What was the sex of the researcher? | Four researchers: Female  One researcher: Male |
| 5 | Experience and education | What are the experiences and education levels of the researchers? | Frist author: expertise in conducting qualitative research, psychosocial oncology.  Second author­: expertise in mixed-method, psychosocial oncology, digital health  Third author: expertise in psychosocial oncology, digital health  Fourth author: expertise in psychosocial oncology and palliative care  Fifth author: expertise in digital health, cancer survivorship, implementation science, mixed-method and intervention research |
| Relationship with participants | | | |
| 6 | Relationship status | Was there a relationship between the researcher and the participants before the training? | No, there was not. |
| 7 | Interviewee’s information about the interviewer | What did the participants know about the researcher (eg, personal goals and reasons for doing the research)? | Participants knew that the researcher was a PhD candidate in health psychology and is doing this research as part of her PhD thesis. |
| 8 | Interviewee characteristics | What characteristics of the interviewer/facilitator were reported (eg, bias, assumptions, reasons, and interests in research)? | At the beginning of each focus group and interview, the participants were informed about the aim, reason, and objectives of the study. |
| Domain 2: Study Design | | | |
| Theoretical framework | | | |
| 9 | Methodological orientation and theory | What methodological orientation was identified to support the study (eg, discourse analysis, ethnography, phenomenology, and content analysis)? | Thematic analysis |
| Sampling | | | |
| 10 | Sampling | How were the participants selected? (eg, purposeful, convenience, consecutive, snowball)? | Purposeful |
| 11 | Approach method | How were the participants reached (eg, face-to-face, telephone, mail)? | Face-to-face and Telephone |
| 12 | Sample size | How many participants were there in the study? | A total of 30 individuals, consisting of 15 PSOP and 15 caregivers were included in the study. |
| 13 | Exclusion | How many people refused to participate or dropped out? Reasons? | Four PSOP refused to participate in the study due to conflicting schedule and lack of interest.  Two caregivers refused to participate due to lack of interest and time.  No participant dropped out of the study after providing consent. |
| Setting | | | |
| 14 | The setting of data collection | Where were the data collected (eg, home, clinic, or workplace)? | **PSOP:**  Focus Groups:  -The first focus group was conducted in person in a conference room within the Macsa Psychosocial and Palliative Care Clinic, located at Fatima Public Hospital.  -The second focus group was held virtually via the Skype videoconferencing platform.  Individual Interviews:  -One interview was conducted in person in a private room in the Macsa Clinic, Fatima Hospital.  -One individual interview was conducted in person in a conference room at the Psychology and Counseling Services Centre, affiliated with the Department of Psychology and Educational Studies at Shahid Beheshti University.  -One individual interview was conducted virtually via phone call.  **Caregivers:**  Individual Interviews:  -Ten interviews were conducted in person in a private room in the Macsa Clinic, Fatima Hospital.  -Five individual interviews were conducted virtually via phone call. |
| 15 | Presence of nonparticipants | Was there anyone else other than the participants and the researchers? | No, there was not. |
| 16 | Description of the sample | What are the important characteristics of the sample (e.g., demographic data, date)? | PSOP: mental healthcare providers specialized in working in an oncology setting.  Caregivers: informal family caregiver of a patient with cancer. |
| Data collection | | | |
| 17 | Interview guide | Were questions, prompts, and guidelines provided by the authors? Were they tested in a pilot study? | Questions and prompts were provided by the first author in collaboration with the second and third author. They were tested in a pilot interview with one PSOP and one caregiver separately. |
| 18 | Repeat interviews | Were repeated interviews conducted? If yes, how many? | No, they were not. |
| 19 | Audio/visual recording | Was audio recording or visual recording used to collect data in the research? | Interviews and focus groups were recorded with a voice recorder. |
| 20 | Field notes | Were field notes taken during and/or after the interview or focus group? | All participants’ responses and researcher observations were recorded. |
| 21 | Duration | How long were the interviews or focus groups? | Focus groups: 120 minutes  Interviews: 45-75 minutes |
| 22 | Data saturation | Was data saturation discussed? | Yes, it was. |
| 23 | Transcripts returned | Were transcripts returned to participants for comment and/or correction? | No, they were not. |
| Domain 3: Analysis and results | | | |
| 24 | Number of data coders | How many data coders coded the data? | First author coded the data, then shared the codes with the second, third and fifth author for their opinion. |
| 25 | Description of the coding tree | Did the authors describe the coding tree? | The titles and subtitles in the results section represent the final coding tree. |
| 26 | Derivation of themes | Were the themes predetermined or derived from the data? | Themes were derived from the data. |
| 27 | Software | If any, what software was used to manage the data? | The data were managed using MAXQDA. |
| 28 | Participant control | Did participants provide feedback on the findings? | No, they did not. |
| Reporting | | | |
| 29 | Quotations provided | Are participant quotes cited to illustrate themes/findings? Is each quote identified, eg, by participant number? | Yes, participants quotes are provided in the table 5 and 6. |
| 30 | Data and findings consistent | Was there consistency between the data presented and the findings? | Yes, there was. |
| 31 | Clarity of main themes | Are the main themes clearly presented in the findings? | Yes, they are. |
| 32 | Clarity of subthemes | Is there a description of the different cases or a discussion of minor issues? | Yes, there is. |
